# 1771. End-of-Life Use of Antibiotics in Pediatric Patients

**DOI:** 10.1093/ofid/ofac492.1401

**Published:** 2022-12-15

**Authors:** Patrick LLoyd, Jacquie Toia, Hannah Lust, Emily Shteynberg, Anders Newman, Sameer Patel

**Affiliations:** Children's Hospital Omaha, Omaha, Nebraska; Ann & Robert H. Lurie Children's Hospital in Chicago, Chicago, IL; Ann & Robert H. Lurie Children's Hospital in Chicago, Chicago, IL; Northwestern University, Chicago, Illinois; University of California San Francisco, San Francisco, California; Ann and Robert H. Lurie Children's Hospital, Chicago, Illinois

## Abstract

**Background:**

Antibiotic use at the end of life is common in adult patients but little is known about children. While important to treat and prevent infection in these vulnerable populations, unnecessary antibiotic exposure may result in adverse events and selection of antimicrobial resistant organisms. The purpose of this study was to describe clinical features of pediatric patients who receive antibiotic use at the end of life, quantify antibiotic utilization, and determine indications for antibiotic use.

**Methods:**

We performed a retrospective review of antibiotic use in the last 30 days of life who had palliative care consultation at a tertiary care pediatric hospital from June 2012 to February 2020. Demographic characteristics, primary diagnosis, do not resuscitate (DNR) status, and hospice status were assessed. Days of antibiotic therapy were recorded.

**Results:**

Of the 151 patients identified, 67 (44%) were female, and the median age at death was 3.5 years (IQR: 0.50-13.8 years). Of the causes of death, 133 (88%) had chronic progressive illness, 13 (8.7%) had a chronic static illness, 5 (3.3%) and had an acute illness. The most common primary diagnoses were oncologic (n=56, 37%), cardiac (n=32, 21%), genetic metabolic (n=19, 13%), and neurological (n=16, 11%). DNR status was recorded for 109 (72%) patients, and 11 (7.2%) had documented hospice status. Antimicrobial use was most common for a known organism (n=75, 50%), followed by no documented indication (n=33, 22.8%), empiric therapy (n=31, 20.5%), and prophylaxis only (n=2.7%). Of the 2126 total days of therapy of antibiotic use, the most common agents were vancomycin (n=379), meropenem (n=254), ceftazidime (n=171), and cefepime (n=143). The median number of unique antibiotics received in the last 30 days of life was 2 (IQR 0-5).

**Conclusion:**

Antibiotic exposure was common in pediatric patients at the end of life, particularly for vancomycin and meropenem, even without documented infection. Opportunities for future interventions include better discussion of goals of care between providers and parents and risk of adverse events, particularly if suspicion of infection is low.

**Disclosures:**

**All Authors**: No reported disclosures.

